# The Practical Value of Blood-Counts

**Published:** 1903-09

**Authors:** William Gordon

**Affiliations:** Physician to the Devon and Exeter Hospital.


### THE PRACTICAL VALUE OF BLOOD-COUNTS.

BY

WILLIAM GORDON, M.A., M.D. Cantab.,

*Physician to the Devon and Exeter Hospital.*

THE importance of recent work on blood-counts, and the rather fragmentary information afforded by all except special works, are my excuse for bringing before you a paper which makes no claim to originality, and whose sole object is to outline the present state of the subject as briefly and clearly as I can.

I would restrict what I have to say to the changes in the numbers and appearances of the blood-corpuscles themselves, leaving out of account those parasites which may inhabit them or exist in the plasma between them; I would omit all names of authorities, as tending to interfere with simplicity of statement; also all points at present under dispute, as apt to obscure the definiteness of facts already established; as well as all those details of methods which can be sufficiently gathered from text-books like Clifford Allbutt's *System of Medicine* or Allchin's *Manual*, merely reminding you that these methods consist in:—

I. The examination of fresh blood under the microscope.

II. The enumeration of red and white corpuscles by the hæmocytometer.

III. The estimation of the hæmoglobin by the hæmoglobinometer.

IV. The examination of dried blood-films stained, say, with eosin and methylene blue. (It will be necessary to remember that eosin is an acid and methylene blue a basic stain, so that red-stained structures are termed oxyphile, and blue-stained structures are termed basophile. Structures staining with both colours, *i.e.*, violet, are called polychromatophile.)

Lastly, I would omit, as far as possible, everything which has at present no practical application.

Before describing what is pathological, it will be well to remind ourselves of what is physiological.

### NORMAL CONDITIONS.

### The Red Corpuscles.

In health the red corpuscles number about five millions per cm. in the adult male, and about four and a half millions in the adult female. At birth they exceed these numbers, nearly amounting to six millions, but the excess vanishes in about a week. 75 per cent. of the red corpuscles measure 7.5 $\mu$. across ($\mu$. being the one-thousandth part of a millimetre), and of the remaining 25 per cent., about half are larger, nearly 9 $\mu$., and half are smaller, nearly 6 $\mu$. A few very small ones, called microcytes, are sometimes found, varying in size from 3.5 $\mu$. to 6 $\mu$. The shape of normal red corpuscles is fairly uniform, and so is their hæmoglobin value. They are faintly oxyphile, and therefore stain pink with a mixture of eosin and methylene blue.

### The White Corpuscles.

In health the white corpuscles vary considerably in number. We may probably take 7,500 per cubic millimetre as the normal average, and 5,000 and 10,000 as the normal limits of variation. Within these limits certain physiological conditions considerably affect their numbers. Thus proteid digestion and violent exercise cause what is spoken of as "physiological leucocytosis," *i.e.*, an increase in number which may amount to 33 per cent. Physiological leucocytosis due to these causes probably never raises the numbers of the white corpuscles to above the 10,000 limit; but there are two other causes of leucocytosis which must be regarded as physiological, yet give rise to a much greater increase in numbers, viz., the leucocytosis of childbirth and the leucocytosis of the newly-born. The former begins during pregnancy, reaches its maximum of about 20,000 at parturition, remains high for about a week, and then gradually subsides; the latter may amount to 21,000 just after birth, but falls to nearly normal in about ten days.

In order to estimate the degree of digestive leucocytosis in a given person, the corpuscles must be counted before the first meal of the day and at intervals after it. The leucocytosis begins about an hour after the meal, reaches its maximum in about three hours, and subsides again in about three hours more. It is greatest after a meat meal, less after a mixed meal, and absent after a vegetable meal.

There are five kinds of white corpuscles found in healthy blood, viz., polymorphonuclear cells, eosinophile cells, large hyaline cells, small hyaline cells, and finely granular basophile cells. Their relative proportions have been variously stated. We shall probably not greatly err in stating them thus:—

| | |
|---|---|
| Polymorphonuclears ... | 70 per cent. |
| Eosinophils ... ... ... | 2 ,, |
| Large hyaline cells ... ... | 4 ,, |
| Small hyaline cells ... ... | 24 ,, |
| Finely granular basophiles ... | extremely few. |

In infants the hyaline cells, especially the small hyaline cells, are relatively much more numerous, the polymorphonuclears being correspondingly fewer.

The **Polymorphonuclear cell** measures about 10 $\mu$. in diameter. Its nucleus seems divided into several,—hence the name,—and stains deeply with methylene blue. Its protoplasm is fairly abundant, and is full of fine granules, which stain pink with eosin; hence it is sometimes called a finely granular oxyphile cell. In fresh blood it is amœboid.

The **eosinophile cell** measures about 11 $\mu$. in diameter. Its nucleus is less divided than that of the polymorphonuclear, and stains more faintly with methylene blue. Its protoplasm is fairly abundant, and is loaded with large, strongly-refracting granules, obvious even in fresh blood, but very obvious in stained films, being coloured brilliantly red with eosin; hence the name, eosinophile; it is also called the coarsely granular oxyphile cell. It is amœboid in fresh blood.

The **large hyaline cell** (sometimes also called large mononuclear, or large lymphocyte) may measure 12 $\mu$. in diameter. Its nucleus is single, oval, or notched, staining moderately with methylene blue. Its protoplasm is abundant, and, as its name implies, free from granules, staining faintly blue. It is also amœboid.

The **small hyaline cell** (sometimes called small mononuclear, or small lymphocyte) is about the size of an average red corpuscle, or 7.5 $\mu$. in diameter. Its nucleus is single, large and round, almost filling the cell. The protoplasm is a mere ring. The whole cell stains deeply with methylene blue.

The **finely granular basophile cell** is the smallest and rarest. Its nucleus is much divided, and stains slate colour with methylene blue. Its protoplasm stains faintly, the granules deeply, with the blue.

### PATHOLOGICAL CONDITIONS.

### The Red Corpuscles.

In disease the red corpuscles may occasionally be increased in **number**: thus in the algid stage of cholera six and a half millions per cubic millimetre have been found; but much more commonly they are reduced in number, their reduction constituting one chief feature of anæmia. Anæmia may be taken in terms of the red corpuscles, to begin at 80 per cent., to be decided at 65 per cent., grave at 50 per cent., very grave at 35 per cent., and fatal at 7.5 per cent.

The **hæmoglobin value** of the red corpuscles may be occasionally increased, especially in pernicious anæmia; but it is far more commonly decreased, as in chlorosis, and the so-called secondary anæmias. Anæmia may be taken, in terms of the hæmoglobin, to begin at 80 per cent., to be average at 60 per cent., intense at 40 per cent., and extreme, with imminent danger to life, at 16 per cent.

The **size** of the red corpuscles varies in disease more than in health. Microcytes are more common in anæmia than in health, and megalocytes are found as well : megalocytes measure from 9.5 to 14 $\mu$. across. A very small darkly-coloured microcyte, called Eichhort's corpuscle, has no special significance.

The **shape** of the red corpuscles may be greatly altered. Poikilocytes are red corpuscles which, instead of being circular, are oval, pear-shaped, racket-shaped, or covered with many irregular projections.

**Lacunæ** may appear in the stroma, indicating degeneration. Their **reaction to stains** may alter : instead of staining pink with a mixture of eosin and methylene blue, they may stain violet, *i.e.*, become polychromatophile, or darkly purple-staining granules may appear in their stroma, as in lead poisoning.

**Nucleated red corpuscles** may make their appearance : these are of two kinds, viz., normoblasts, which are almost the size of normal red blood-corpuscles, and megaloblasts, which may measure 16 $\mu$. across. The nuclei stain blue with a mixture of eosin and methylene blue, and the stroma may be either pink or violet. Both may be irregular in shape, and are then called poikiloblasts.

It should be clearly borne in mind that very severe anæmia from any cause may be associated with the presence of poikilocytes, megalocytes, normoblasts, vacuolated corpuscles, and polychromatophiles—also poikiloblasts.

In diseases associated with active removal of hæmoglobin from the red cell, as in paroxysmal methæmoglobinuria (during the attack), the faint outlines of dichromised corpuscles are seen, and named "**shadow-corpuscles.**"

### The White Corpuscles.

In disease the leucocytes may be occasionally reduced in number : this condition, which is called **leucopenia**, is found markedly in typhoid fever ; but much more commonly they are increased in number : this condition, known as "**pathological leucocytosis,**" may be so marked that 100,000 leucocytes are found in each cubic millimetre, as in some cases of pneumonia and of empyema.

The relative proportions of the various forms of white corpuscles may remain unaltered in pathological leucocytosis, as in some cases of sarcoma, but usually, as in abscess cases, the chief increase is found in the polymorphonuclears, which may amount to 95 per cent. of all leucocytes. Less commonly, as in some cases of rapidly-growing sarcoma, and in the lymphatic form of leucocythæmia, as well as in the leucocytosis of infants from any cause, the hyaline cells are chiefly increased: in leucocythæmia they may amount to 95 per cent.

The large hyaline cells (or large mononuclears) may be relatively increased to over 12 per cent. of the white corpuscles present, and this appears to be an important fact in malaria. The eosinophiles may be relatively increased (even to 68 per cent.), and this increase is of importance in trichinosis and other parasitic diseases. New forms of leucocytes may make their appearance.

**Myelocytes** are very large cells, measuring even 20 $\mu$. in diameter. The nucleus of the myelocyte is single, oval, or lobed, staining moderately with methylene blue. The protoplasm is abundant, stains faintly with the blue, and contains granules which stain red with eosin. These granules may be fine or coarse; the coarsely granular cells are called **eosinophile myelocytes.** The myelocytes are not amœboid; they rarely occur except in spleno-medullary leucocythemia, and when they do occur in other diseases they are very few in number. In leucocythemia they may amount to 50 per cent. of all leucocytes. The eosinophile myelocyte is said to be peculiar to spleno-medullary leucocythemia.

**Mast cells** are basophile cells with large irregular granules, staining dark violet with methylene blue; their significance is doubtful, except that they are never found in health: they may measure as much as 20 $\mu$. in diameter.

### APPLICATION OF THE FOREGOING.

What can we learn from the observation of these alterations in disease?

#### 1.—Suppuration.

The presence of leucocytosis of a certain degree may frequently be of the greatest value in the diagnosis of abscess,—apparently of abscess anywhere,—and its degree, or its absence, may give useful prognostic indications.

The chief observations on this point have been made in cases of **appendicitis**, and the outcome for that disease may be summarised thus:—

1. A count of 20,000 or over in the first two or three days strongly suggests general peritonitis.
2. A count of 20,000 or over at the end of the first week almost certainly means an abscess.
3. Leucocytosis to a less extent is doubtful in significance.
4. Absence of leucocytosis means one of three things—
   (*a*) Fulminating appendicitis where the resistance of the body is overwhelmed.
   (*b*) A very mild case.
   (*c*) An abscess enclosed within thick walls.
5. After operation, a leucocytosis which persists beyond the third or fourth day means probably either pockets of pus or peritonitis.
6. Leucocytosis should not be relied on alone; but, taken with other signs and symptoms, is often an invaluable help: it affords earlier indications of pus than surface œdema, sweats or diarrhœa.

As regards prognostic indications—

Absence of leucocytosis in presence of obviously septic appendicitis is of bad augury.

Absence of leucocytosis with mild early symptoms is of good augury.

Absence of leucocytosis with obvious abscess means the presence of a thick wall and absence of extension.

Increasing leucocytosis indicates extending infection.

Diminishing leucocytosis indicates diminishing toxicity or walling of an abscess.

My experience would lead me to strongly recommend the observation of the blood as a routine measure in all cases of appendicitis, even in the rough way of mere inspection of a fresh drop of blood under the microscope. A large leucocytosis is very obvious in such a preparation, and has on several occasions guided me in doubtful and discussed cases to a correct diagnosis of pus. Hæmocytometers are not always at hand, and everyone does not possess one, nor has everyone the time needed for a regular blood-count ; but very few medical men are nowadays without a microscope, yet few realise how obvious a leucocytosis of 20,000 is in a fresh specimen of blood, when compared with a similar specimen from the finger of a healthy person. An observation sufficient to determine this occupies about five minutes, and a blood-film may be stained with eosin and methylene blue in about another five minutes.

**Abscess of the brain** has given me a very decided leucocytosis : this will probably be a valuable distinction from cases of tumour, as the following instance suggests.

Some time ago a small boy was admitted under my care in this hospital with headache, sickness, double optic neuritis, low temperature and pulse-rate, drowsiness, and paralysis of the left third cranial nerve, with paresis of right arm and right lower half of face. He had had for years a discharge of pus from the left ear, but this had recently stopped. I think no one will wonder that I asked my colleague, Mr. Harris, to operate for a probable large cerebral abscess in the left tempero-sphenoidal lobe. But there was no leucocytosis. No abscess was found, but after death, some weeks subsequently, a tumour was discovered growing in the tempero-sphenoidal lobe, just over the diseased ear. In another such case absence of leucocytosis would probably guide one to diagnose tumour rather than abscess, though still, no doubt, the proper course would be to trephine and explore, because leucocytosis would probably be absent if the pus were thickly walled in.

**Empyema.**—I have several times suspected that an effusion into the chest was purulent on account of a considerable leucocytosis, and found myself mistaken. But after seeing the tremendous leucocytosis which empyema produces, one will be little likely to fall into that error again. Osler has recorded a case where the white corpuscles amounted to 115,oco per cubic millimetre. It is very interesting to note that serous tubercular pleurisy seems to produce no leucocytosis at all. A blood-count has enabled me to exclude empyema before the exploring syringe showed that the fluid was serous.

**Liver abscess,** according to several observers, may be diagnosed by the presence of leucocytosis from other diseases of that organ. Probably the degree of leucocytosis varies with the organ in which the abscess lies (thus its degree is different in appendicular abscess and empyema). Another useful test for suppuration is the so-called iodine reaction of the white corpuscles.

### 2.—Blood Diseases.

Turning next to so-called "Blood diseases."

**In chlorosis** the hæmoglobin is reduced more than the red corpuscles; the red corpuscles rarely fall below two millions. Megalocytes are not common, megaloblasts absent or very rare. Leucocytosis does not occur.

**In pernicious anæmia** the red corpuscles are generally more reduced than the hæmoglobin; the red corpuscles frequently fall below one million. Megalocytes are always present; megaloblasts are generally present and in greater numbers than the normoblasts. Leucocytosis may occur late, but leucopenia is the rule. A few myelocytes may be found.

**In splenic anæmia** the hæmoglobin falls more than the red corpuscles; the red corpuscles may fall even to one million. Megalocytes do not occur, nor does leucocytosis. These characters are of immense importance as distinguishing this disease, in which splenectomy usually cures, from leucocythæmia in which it is invariably fatal.

**In Hodgkin's disease** the blood is normal in the early stages, and, later, the changes are not pronounced.

**In secondary anæmias** the hæmoglobin falls faster than the red corpuscles; the blood shows no special features, except those of severe anæmia when it occurs.

**In spleno-medullary leucocythemia** red cells and hæmoglobin generally fall together; the red cells rarely fall below two millions. Megalocytes may occur. Normoblasts are constantly present, even when anæmia is slight and their occurrence with so little anæmia is said to be peculiar to the disease. Megaloblasts are generally present, but in smaller numbers than the normoblasts. The leucocytes are enormously increased—half a million is not uncommon; but more important than the number is the presence of a large proportion of myelocytes—even 50 per cent., which occurs in no other disease. Eosinophile myelocytes are said to be only found in spleno-medullary leucocythemia.

**In lymphatic leucocythæmia** the hæmoglobin falls about *pari passu* with the red cells; the red cells, however, almost always fall further than in spleno-medullary leucocythæmia. Normoblasts and megaloblasts are extremely rare, and if found are few. The leucocytes are greatly increased, but not so greatly as in the spleno-medullary disease; they vary from forty thousand to a quarter of a million Large and small hyaline cells are the predominant form of leucocytes, constituting sometimes 95 per cent. of those present ; myelocytes are rarely found, and if found are few. These characters do not always distinguish from cases of sarcoma.

**In hæmorrhagic blood diseases** the blood characters are seemingly just those of post-hæmorrhagic anæmia. Hæmoglobin falls faster than red cells. Megaloblasts are always fewer than normoblasts if present. Leucocytosis is usual, and generally chiefly affects the polymorphonuclears.

### 3.—Helminthiasis.

The worm-parasitic diseases have the curious characteristic of often increasing the relative number of eosinophiles producing a condition known as "eosinophilia." It is at once obvious in a stained film.

**In trichinosis** this increase may actually amount to 68 per cent. of a leucocytosis of 20,000, and is of decided value in diagnosis.

**In anchylostoma** 72 per cent. have been observed. Such a count in a case of very severe anæmia in the tropics would lead to a search in the stools for the characteristic ova.

**In tænia mediocanellata** 34 per cent have been seen.

**In filariasis, bilharzia, ascarides and oxyuris,** counts of about 20 per cent. have been recorded.

### 4.—Other Causes of Eosinophilia.

But *eosinophilia* is not confined to worm-diseases. In *emphysema* and in *the attacks of true asthma* it has been discovered, and it is said that by its means true asthma can be distinguished from so-called cardiac and renal asthma.

In some functional nervous diseases as *chorea*, *hysteria*, and *neurasthenia*, and in some skin diseases as *pemphigus*, *psoriasis*, *dermatitis herpetiformis* and *leprosy*, eosinophilia exists to a moderate degree.

**5.--Acute Specifics.**

In certain *acute specific diseases*, the characters of the blood-corpuscles may be of great value.

In **typhoid** leucopenia exists, and may be marked. This distinguishes the disease from diseases like gastro-enteritis, in which there is leucocytosis; leucocytosis, however, sets in rapidly when a complication occurs in typhoid, as hemorrhage or perforation.

In *typhus*, *influenza*, *measles*, *tuberculosis* and *malaria*, there is usually no leucocytosis. On the other hand, leucocytosis occurs in *pneumonia*, *scarlet fever*, *small-pox*, *diphtheria*, *erysipelas*, *whooping cough*, *acute rheumatism*, *pyæmia*, *septicæmia*, and *plague*. The diagnostic importance of these differences is obvious.

Its absence in uncomplicated **tuberculosis** is a point of considerable value. In **malaria** the usual low leucocyte count seems to be of undoubted value; but even more striking appears the diagnostic value of a relative increase, which occurs in the large mononuclear hyaline cells to 12 per cent. and over. This increase of large mononuclears has been found in 90 per cent. of cases, whilst malaria parasites could only be demonstrated in 17 per cent.

In **pneumonia** the leucocytosis is immense, and near the crisis may reach 100,000. The rules governing it seem very like some of those connected with the leucocytosis of appendicitis: thus a malignant pneumonia may have no leucocytosis, and the absence of leucocytosis in a very severe case is of bad omen. Apart from cases so bad as this, the degree of leucocytosis is usually in proportion to the severity of the case; a very abundant leucocytosis at the time of crisis is said to be a good sign.

In **whooping cough** the very marked leucocytosis is said to be a useful diagnostic point before the whoop develops.

### 6.—Young Children.

In examining the blood of very young children it is important to remember that—

I. Severe anæmia may result from trifling causes.
II. That leucocytosis may also result from slight causes,
III. And is apt to be a lymphocytosis.
IV. That the spleen is apt to enlarge in any severe anæmia.
V. That nucleated red cells are more commonly found.
VI. That hereditary syphilis and rickets, cause some of the severest anæmias;
VII. That in them tuberculosis does cause leucocytosis.

### 7.—Malignant Disease.

With regard to malignant disease, opinions seem to differ as to the value of a blood examination. I have found very marked leucocytosis in some cases of sarcoma. The absence of digestive leucocytosis in cases of cancer of the stomach becomes less useful diagnostically when we consider that other gastric diseases, such as chronic gastritis, may give rise to it.

In conclusion, I may say that I feel I have placed before you a very rough *résumé* of a wide subject; but if I have satisfied you that the subject is a very important one, I shall have been justified in doing so.

